# Editorial: NK-Myeloid Cell Interactions in the Tumor Microenvironment: Implications for Cancer Immunotherapy

**DOI:** 10.3389/fimmu.2021.718844

**Published:** 2021-07-01

**Authors:** Erik Wennerberg, Andreas Lundqvist, Yumeng Mao, Dimitrios Mougiakakos

**Affiliations:** ^1^ Division of Radiotherapy and Imaging, The Institute of Cancer Research, London, United Kingdom; ^2^ Department of Oncology-Pathology, Karolinska Institutet, Stockholm, Sweden; ^3^ Science for Life Laboratory, Department of Immunology, Genetics and Pathology, Uppsala University, Uppsala, Sweden; ^4^ Department of Medicine 5 for Hematology and Clinical Oncology, Friedrich Alexander University (FAU), Erlangen, Germany

**Keywords:** NK cells, myeloid cells, dendritic cells, MDSCs (myeloid-derived suppressor cells), cancer immunotherapy, platelets, cytokines

Over the last decades, immunotherapy has revolutionized cancer treatment. The emergence of immune checkpoint inhibitors (ICI) targeting PD-1 and CTLA-4, has rendered many aggressive cancers treatable and even curable. However, ICI often fails to generate sustained responses, particularly in patients with low or absent pre-existing T cell immunity. In order to turn these “cold” tumors “hot”, initiation of *de novo* tumor-specific immune responses is required, a process which is dependent on the actions of innate immune cells, and whose infiltration and function is highly impacted by the composition of the tumor microenvironment (TME).

Natural killer (NK) cells are innate lymphoid cells that play essential roles in cancer immunosurveillance and anti-tumor immunity due to their unique ability to identify and kill tumor cells by recognizing missing-self and induced stress ligands. The cytolytic potential of NK cells can be harnessed for treatment of advanced cancers through adoptive cell transfer therapies and by augmenting their function and persistence *in vivo*. Recently discovered bi-directional crosstalk between NK cells and certain subsets of dendritic cells (DCs), which are the key orchestrators of initiating, maintaining and regulating anti-tumor immunity, has shed new light on the role of NK cells in shaping adaptive immune responses in cancer. Importantly, emerging evidence of how NK cell function is suppressed by myeloid cells in the TME, warrants a re-examination of how cancers evolve to specifically evade NK cell killing *via* recruitment and polarization of tumor-associated macrophages (TAMs) and myeloid-derived suppressor cells (MDSCs).

Here, we present a collection of reviews, mini-reviews and original research articles which discern the interactions between NK cells and myeloid cell subsets, how their crosstalk regulates the anti-tumor immune response, and how it can be manipulated to potentiate cancer immunotherapies.

## NK-DC Interactions

Recent work has highlighted that NK cells and DCs, and in particular conventional type 1 DC, (cDC1) engage in an intercellular crosstalk to coordinate adaptive immunity against cancer. Furthermore, this crosstalk has also been linked to increased survival and responses to anti-PD-1 immunotherapy in patients with metastatic melanoma. In this edition, Peterson and Barry review recent findings on the role of NK cells and cDC1s in protective immune responses to cancer and immunotherapy, as well as current therapies targeting this NK cell–cDC1 axis. In addition, Bödder et al. discuss the function of cDC1s and NK cells, their bidirectional crosstalk and potential strategies to improve cancer immunotherapy. In the TME, different DC subsets can display tumor-promoting or -inhibiting characteristics. To this end, Jacobs et al. discuss recent findings on the interaction of DCs and NK cells under physiological conditions and within the TME. In addition, they discuss potential strategies to overcome the immunosuppressive outcome of the DCs and NK cells interaction within the TME.

## NK-Platelet Interactions

Emerging evidence reveal that the interaction between platelets and tumor cells in peripheral blood is an important factor to support dissemination of cancer. Platelets can also produce high levels of TGF-β to support an immunosuppressive TME. Schmied et al. review the interplay between platelets and NK cells in solid tumors and also discuss how these could apply to hematological malignancies. They furthermore explore the possible implications for NK cell therapy in patients with solid tumors and patients who depend on frequent platelet transfusions. The activating receptor NKG2D induces NK cell-mediated killing of tumor cells by recognition of stress-induced ligands. Platelets can shield tumor cells directly from engagement of NK cell-mediated attack by cleaving such stress-induced ligands from tumor cells. To this end, Maurer and de Andrade discuss the underlying mechanisms of NK cell control by platelets with a focus on NKG2D and its ligands, providing a perspective to exploit these pathways with novel immunotherapeutic approaches.

## NK-MDSC Interactions

MDSC is a heterogeneous population that contains a range of immature cells with potent inhibitory functions against anti-tumor immunity. MDSC can be divided broadly into monocytic and polymorphonuclear (PMN) subsets according to the lineage origin and cell morphology. A wealth of literature evidence demonstrates that MDSC can regulate NK cell function in health and disease. In this special issue, Pelosi et al. investigated the effects of G-CSF mobilization treatment on NK cells and PMN-MDSC in healthy individuals. The authors demonstrated that G-CSF mobilization reduced the cytolytic functions of NK cells against a leukemia cell line. Marked changes of gene expression profiles were observed in NK cells and PMN-MDSC isolated from G-CSF mobilized individuals, as compared to the non-mobilized counterparts. PMN-MDSC showed prolonged survival and better migration in response to chemo-attractants. This study provides an important data source to dissect the immune regulatory effects of G-CSF mobilization during leukemia treatment. In a separate study, Gallazzi et al. reported elevated exhaustion markers on peripheral NK cells from prostate cancer patients, as compared to the healthy controls. Similar changes were observed when NK cells were treated with TGF-β or IL-6. Using *in vitro* co-culture models, the authors showed that these patient-derived NK cells could activate the inflammatory responses in endothelial cells and reshape the functions of monocytes and macrophages. Altogether, this paper highlights the functional interactions between NK cells and other cell compartments in the prostate cancer microenvironment.

## NK Cell Crosstalk in Cancer Therapy

Adoptive NK cell transfer for treating hematological malignancies led to promising results and was followed by a substantial number of studies in solid tumors. Genetically modified NK cells carrying tumor antigen-targeting chimeric antigen receptors (CARs) are currently under clinical investigation. Overall, clinical efficacy has been limited in particular in solid tumors thus highlighting the need to better understand the hurdles within the permissive TME. Myeloid cells are characterized by an exceptional plasticity and represent a key cell population within the TME. They have been shown to readily obtain tumor-promoting properties as M2-type macrophages, TAMs, and MDSCs. Zalfa and Paust comprehensively describe the mechanisms leading to MDSC accumulation (e.g., induction by cytokines or chemokine-mediated recruitment) in cancer and how those heterogeneic cells interact with NK cells thereby limiting their anti-tumor activity. Finally, the authors offer an extensive overview on strategies to interfere with MDSCs and to thereby harness the efficacy of NK cell-based immunotherapies. Tumino et al. look at the interaction of NK cells and MDSCs from the perspective of the allogeneic hematopoietic stem cell transplantations (allo-HSCTs). In fact, allo-HSCT remains one of the most successful immunotherapies and is for some malignant diseases the only therapy with a curative potential. The therapeutic success is based on the so-called graft versus leukemia (GvL) effect and donor NK cells are important mediators of the GvL. The authors explain how reconstituting monocytic or neutrophilic MDSCs counteract the NK cells’ GvL activity especially during the early post-transplant period. Furthermore, they describe pharmacological interventions that could reduce the immunosuppressive activity of MDSCs thus improving the outcome of allo-HSCTs. Carnevalli et al. focus in their review on current and future therapeutic approaches to target the detrimental interaction between myeloid cells (including MDSCs and tolerogenic macrophages) and (anti-tumor) NK cells. The strategies that are introduced comprise amongst others blockade of inhibitory checkpoints (on myeloid cells), triggering of co-stimulatory receptors (on NK cells), application of myeloid-cell inhibitors (e.g., CSF1R inhibitors), and interference with intracellular myeloid cell signaling (e.g., STAT3). Finally, Gaggero et al. give an overview on cytokines that antagonize (e.g., TGF-β) and that promote (e.g., IL-15) NK-cell function. They emphasize how remodeling of the TME cytokine milieu could skew the balance between anti-tumor reactivity and tumor tolerance towards the former. They also speculate about the generation of genetically engineered CAR NK cells that carry cytokine signaling elements or dominant negative cytokine receptors and are therefore resistant towards the TME, which would ensure their longevity and persistence.

## Author Contributions

All authors listed have made a substantial, direct, and intellectual contribution to the work and approved it for publication.

## Conflict of Interest

The authors declare that the research was conducted in the absence of any commercial or financial relationships that could be construed as a potential conflict of interest.

